# Guanylyl Cyclase-cGMP Signaling Pathway in Melanocytes: Differential Effects of Altered Gravity in Non-Metastatic and Metastatic Cells

**DOI:** 10.3390/ijms21031139

**Published:** 2020-02-08

**Authors:** Krassimira Ivanova, Ruth Hemmersbach

**Affiliations:** Institute of Aerospace Medicine, Department of Gravitational Biology, German Aerospace Center (DLR), Linder Hoehe, 51147 Cologne, Germany; ruth.hemmersbach@dlr.de

**Keywords:** human melanoma cells, cGMP signaling, NO, sGC, natriuretic peptides, GC-A/GC-B, clinostat, microgravity, hypergravity

## Abstract

Human epidermal melanocytes as melanin producing skin cells represent a crucial barrier against UV-radiation and oxidative stress. It was shown that the intracellular signaling molecule cyclic guanosine-3′,5′-monophosphate (cGMP), generated by the guanylyl cyclases (GCs), e.g., the nitric oxide (NO)-sensitive soluble GC (sGC) and the natriuretic peptide-activated particulate GC (GC-A/GC-B), plays a role in the melanocyte response to environmental stress. Importantly, cGMP is involved in NO-induced perturbation of melanocyte–extracellular matrix interactions and in addition, increased NO production during inflammation may lead to loss of melanocytes and support melanoma metastasis. Further, the NO-sensitive sGC is expressed predominantly in human melanocytes and non-metastatic melanoma cells, whereas absence of functional sGC but up-regulated expression of GC-A/GC-B and inducible NO synthase (iNOS) are detected in metastatic cells. Thus, suppression of sGC expression as well as up-regulated expression of GC-A/GC-B/iNOS appears to correlate with tumor aggressiveness. As the cGMP pathway plays important roles in melanocyte (patho)physiology, we present an overview on the differential effects of altered gravity (hypergravity/simulated microgravity) on the cGMP signaling pathway in melanocytes and melanoma cells with different metastatic potential. We believe that future experiments in real microgravity may benefit from considering cGMP signaling as a possible factor for melanocyte transformation and in medication.

## 1. Introduction

**Figure 1:**
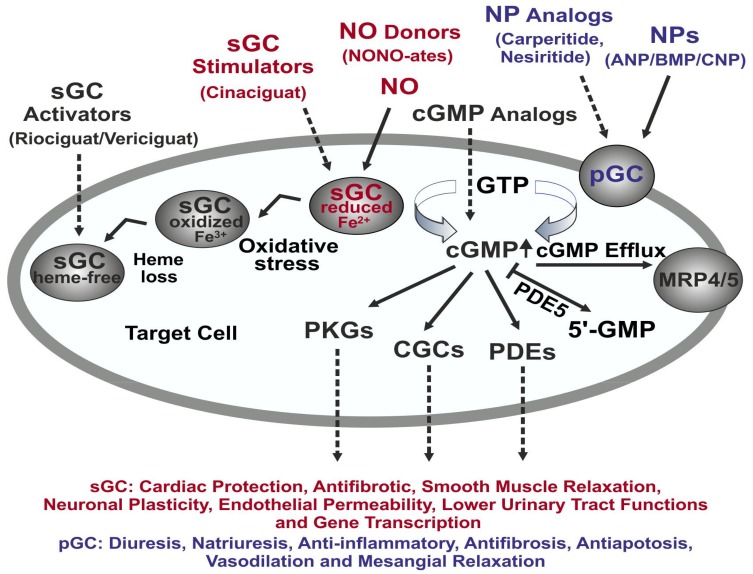
Guanylyl cyclase-cGMP signaling pathway. The guanylyl cyclase (GC) catalyzes the production of cGMP from GTP. NO, NO donors, and sGC stimulators activate the soluble GC (sGC), a heterodimer with a heme prosthetic group in its reduced iron (Fe^2+^) state, whereas the sGC activators activate the heme-free sGC, independent of NO. The natriuretic peptides (NPs) as well as NP analogs activate the particulate GC (pGC). The membrane-permeable cGMP analogs lead directly to an increase of the intracellular cGMP level. Cyclic GMP binds to cGMP-dependent protein kinases (PKGs), cGMP-gated ion channels (CGCs), and cyclic nucleotide-regulated phosphodiesterases (PDEs), which modulate several downstream cellular and physiological responses. PDE5 is a cGMP-specific PDE that inhibits the degradation of cGMP to 5′-GMP. MRP4/5 act as exporters for cGMP. Abbreviations are: ANP, atrial natriuretic peptide; BNP, B-type natriuretic peptide; CNP, C-type natriuretic peptide; MEP4/5, multidrug resistance proteins 4 and 5; NO, nitric oxide.

Cyclic guanosine-3′,5′-monophosphate (cGMP or cyclic GMP) is an important intracellular signaling molecule that regulates several (patho)physiological processes in multiple cell types. It is involved in vascular muscle cell relaxation, reducing platelet aggregation, neuronal transmission, cell growth and survival, vision, and cancer [1,2,3,4]. Cyclic GMP may also play a role in the metabolic and energetic signaling [5]. It is generated by two classes of guanylyl cyclases (GCs), namely the cytosolic or soluble GC (sGC) and the transmembrane-spanning or the particulate GCs (pGCs) (Figure 1). The sGC is the intracellular receptor for nitric oxide (NO). There are three genetically distinct isoforms of nitric oxide synthase (NOS) that catalyze the synthesis of NO from L-arginine: the constitutively expressed and calcium-activated endothelial isoform (eNOS), the neuronal (nNOS), and the cytokine- or endotoxin-induced, calcium-independent isoform (iNOS) that is expressed in response to inflammatory defense against pathogens [6]. NO produced by nNOS is an important neurotransmitter, whereas NO produced by eNOS acts as a paracrine signal predominantly in response to hypoxia and/or mechanical stimuli in the vasculature.

Mammals express four sGC cyclase isoforms (α_1_, α_2_, β_1_, β_2_,). The best characterized heterodimer in humans consisting of an α_1_ and a β_1_ subunit is expressed in most cell types and tissues. Each sGC α and β subunit of the heterodimer has four domains: the N-terminal heme-containing NO/oxygen-binding (H-NOX) domain, a Per/ARNT/Sim (PAS) domain, a coiled-coil signaling helix (CCs), and the catalytic domain [7,8,9]. Only the β subunit binds the heme prosthetic group containing iron. The heme moiety of the sGC in its reduced iron (Fe^2+^) state is bound to the β subunit H-NOX domain through the axial ligand histidine 105 (His 105), building a penta-coordinate hystidyl–heme complex. The primary activation of sGC is the binding of NO to the sixth position of this complex, followed by a subsequent breaking of the bond between the axial His 105 and Fe^2+^ to form a penta-coordinate nitrosyl–heme complex [10,11]. The binding of NO to the heme causes a structural change in the H-NOX domain, which is allosterically transmitted to the catalytic domain, probably leading to a relief of the initial inhibition of the catalytic domain in the absence of NO [12]. In addition, sGC can be desensitized under condition of oxidative stress (presence of reactive oxygen species, ROS), which is believed to be causal in the pathogenesis of many cardiovascular diseases. ROS are capable of oxidizing the heme Fe^2+^ to Fe^3+^, resulting in the loss of the heme group from the oxidized sGC. The heme-free form of sGC is dysfunctional as it is insensitive to NO. Thus, oxidation or nitrosylation of critical cysteine residues may lead to enzyme inactivation [13,14].

Importantly, several members of the GC family are known as drug targets because their activators are associated with disease of the cardiovascular, skeletal, intestinal, or visual systems. For example, the NO-sensitive sGC is an attractive target for cardiopulmonary disorders like angina pectoris, heart failure (HF), as well as peripheral and pulmonary arterial hypertension (PAH) [15]. Focusing on sGC as a therapeutic target, two classes of sGC agonists (sGC “stimulators” and “activators”) have been discovered (Figure 1) [16,17]. The stimulators, e.g., riociguat [18,19] and vericiguat [20], bind to sGC with heme in its reduced (Fe^2+^) state and potentiate the NO–sGC–cGMP signaling. Although the sGC stimulators can increase the enzyme activity in the absence of NO, they can also synergize with NO and therefore are powerful amplifiers of endogenous NO signaling. Riociguat is approved for the treatment of PAH and chronic thromboembolic pulmonary hypertension. In addition, preclinical and clinical evidence include further antifibrotic effects of the sGC stimulators [21,22]. The second class of the sGC agonists, known as sGC activators (e.g., cinaciguat and ataciguat), are able to bind to the heme pocket of sGC and may activate only the heme-free sGC, independent of NO [23]. Preclinical studies with sGC activators revealed beneficial effects of these drugs in various pathologies, including PAH and acute HF, as well as renoprotective effects [23,24].

In contrast to sGC, the mammalian pGC-A or GC-A and pGC-B or GC-B, consisting of an extracellular ligand binding domain, a transmembrane spanning region, an intracellular protein kinase-like homology, and GC catalytic domains, exist as homodimers [25,26]. GC-A is highly expressed in kidney, lung, adrenal, vasculature, brain, liver, endothelial, and adipose tissues [27,28], as well as at low levels in the heart [29]. GC-B is expressed in brain, lung, bone, heart, and ovary tissue but also in fibroblasts and vascular smooth muscle cells [25]. GC-A and GC-B function as the transmembrane receptors for the natriuretic peptides. Atrial natriuretic peptide (ANP) and B-type natriuretic peptide (BNP), which are secreted from the heart atrium and the brain, signal through GC-A, while C-type natriuretic factor (CNP) activates GC-B. In addition, urodilatin (URO), which is synthesized in kidney tubular cells and secreted luminally, is also a native activator of GC-A [30]. GC-A and GC-B B are under basal conditions highly phosphorylated, and dephosphorylation inhibits the receptors [31,32,33].

For GC-A as a target, the drugs Carperitide and Nesiritide have been approved for the treatment of congestive HF, and for the chimeric natriuretic peptide CD-NP, positive therapeutic effects in a clinical study have been reported [34,35]. Recently, a novel GC-A activator, CRRL269, was designed, which induces in in vivo experiments an enhanced diuresis, natriuresis, and an increased glomerular filtration rate in comparison with the native GC-A activators BNP and URO [36]. GC-B is the target for drugs in the development of a treatment of skeletal diseases [37].

Effects of cGMP occur through three main groups of cellular targets such as cGMP-dependent protein kinases (PKGs), cGMP-gated ion channels (CGCs), and cyclic nucleotide-regulated phosphodiesterases (PDEs) [1,38], which modulate several downstream cellular and physiological responses. The cGMP-dependent PDEs are involved in controlling the intracellular cGMP concentration as well as in terminating the cGMP signaling. Three members of the mammalian PDE family (PDE1-11), e.g., PDE 5, 6, and 9, have much higher substrate preference for cGMP compared to cyclic adenosine-3′,5′monophosphate (cAMP) as substrate and are therefore considered as “cGMP specific” PDEs [38]. PDE6 expression is restricted to photoreceptor and pineal cells. PDE5 is expressed in vascular smooth muscles, heart, platelets, and low urinary tract organs, whereas PDE9 is expressed in the retinal pigment epithelial cells and in certain neurons [39].

A number of inhibitors of cGMP-dependent PDEs are in clinical use for the treatment of cardiovascular diseases [39]. The PDE-5-selective inhibitors sildenafil (Viagra), vardenafil (Levitra), and tadalafil (Cialis) are involved in the treatment of various vascular diseases including angina pectoris, erectile dysfunction, and in pulmonary hypertension [40,41]. Importantly, clinical studies demonstrate that the use of PDE5 inhibitors may be associated with prostate cancer progression [42]. Finally, the effective intracellular accumulation of GMP is not only dependent on the activities of the GCs and the metabolic degradation by the PDEs, but also on the active export into the extracellular space, e.g., via the multidrug resistance proteins 4 (MRP4) and 5 (MRP5) as selective cGMP exporters [43]. In addition, both MRP4 and MRP5 also export nucleoside analogs that are used in antiviral and anticancer therapy [44].

## 2. Guanylyl Cyclase-cGMP Signaling Pathway in Melanocytes

The human skin is the first barrier protecting internal organs from harmful effects of environmental factors like solar ultraviolet (UV) radiation and mechanical stimuli that may dramatically alter skin homeostasis. Human epidermal melanocytes, which are located in the basal layer of the skin epidermis, function as a crucial barrier against UV radiation as well as oxidative stress by producing melanin within melanosomes. Melanin is thought to filter out UV radiation and scavenge ROS, thereby reducing the UV damage in the cutaneous cells [45]. Importantly, melanin may also generate ROS upon UV radiation if its scavenging capacities are overwhelmed [46]. Such an accumulation of toxic radicals may induce depigmentation (vitiligo) or melanocyte transformation (melanoma) as well as secondary damages in bystander cells [47]. In addition, the strategic position of melanocytes in the skin, their molecular make-up, and dendritic morphology indicate that melanocytes may play a role in the skin immune system [48].

**Figure 2:**
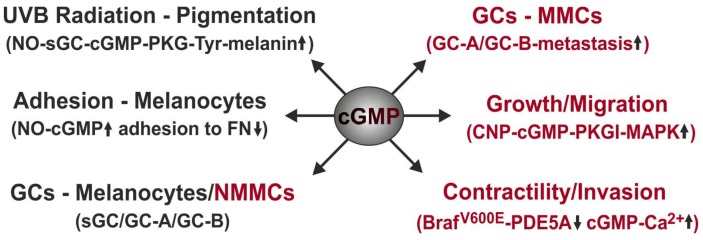
Guanylyl cyclase-cGMP signaling pathway in melanocytes and non-metastatic and metastatic melanomas. cGMP signaling is involved in UVB-induced pigmentation of melanocytes (black color) and in NO-induced reduction of melanocyte adhesion to extracellular matrix (ECM) components like fibronectin (FN). Human melanocytes express GC-A and GC-B but are insensitive to ANP and CNP. Non-metastatic melanoma cells (NMMCs, red color) express the NO-sensitive sGC and the ANP-sensitive GC-A/CNP-sensitive GC-B. Absence of NO-sensitive sGC but upregulated expression and activities of GC-A/GC-B are related to the metastatic potential of melanoma cells (red color). Activation of CNP-GC-B-cGMP-PKGI pathway promotes melanoma growth/migration in a MAPK-dependent manner. The oncogenic BRAF^V600E^ variant induces melanoma cell invasion by downregulating the cGMP-specific PDE5A. Tyr, tyrosinase MMCs, metastatic melanoma cells.

For human epidermal melanocytes, it has been shown that UVB radiation, the physiological stimulus of melanogenesis, acts through the NO-cGMP-PKG pathway, and a constitutive form of NOS is involved in the process [49,50]. We have reported in previous in vitro studies that NO-releasing compounds induce a concentration-dependent reduction in the adhesion of human melanocytes (normal and vitiliginous melanocytes) to extracellular matrix (ECM) components like fibronectin (FN) [51,52]. The effects of NO on the perturbation of the melanocyte–FN interaction were at least partly cGMP dependent in [52]. We further reported that immortalization of normal human and vitiliginous melanocytes via transfection with human papilloma virus 16 (HPV16)-E6 and HPV16-E7 genes does not alter the de novo properties of NO to induce cell detachment from ECM components [53]. Moreover, our results show that normal human melanocytes, vitiliginous melanocytes, and their immortalized counterparts express functional sGC, the prime target for the signaling activity of NO, which is related to the cellular melanin content [53,54]. In addition, normal human melanocytes express GC-A and GC-B but are insensitive to the effects of ANP and BNP. The role of cGMP signaling in human melanocytes is summarized in Figure 2.

## 3. Guanylyl Cyclase-cGMP Signaling Pathway in Melanoma

Metastatic melanoma is responsible for a mortality of about 80% in skin cancer due to its aggressiveness and resistance to existing therapies [55,56]. To efficiently metastasize, invasive melanoma cells need to change their cytoskeletal organization and to alter the contact with the extracellular matrix (ECM) and the surrounding stromal cells. The cutaneous melanoma is a complex genetic disease as a consequence of genetic alterations induced by sporadic mutations as well as by environmental factors. Mutations activating oncogenes and inactivating tumor suppressors may lead to the deregulation of important intracellular signaling pathways and the interaction of melanoma cells with the tumor microenvironment, resulting in a primary tumor progression and eventually metastatic progression [57]. Thus, the characterization of crucial signaling pathways altered in melanoma in comparison to normal and transformed melanocytes may contribute to the development of new and more effective therapeutic approaches.

We have reported differential expression of functional GCs in human melanocytes and melanoma cells [54]. Whereas normal melanocytes and non-metastatic melanoma cells express the NO-sensitive sGC, the melanoma cells do not express functional sGC due to the lack of sGC-β_1_ subunit expression, leading to a loss of NO sensitivity in a control mechanism involving the NO–sGC–cGMP pathway. Moreover, the strong expression and activities of the membrane-bound GC-A/G-CB in the melanoma cells were related to their metastatic potential. The increase of intracellular cGMP concentration in response to CNP was much stronger in metastatic melanoma cells than the respective response to ANP [54], suggesting a role of CNP in the cGMP signaling pathway in melanoma cells. As CNP can be secreted from endothelial cells in response to inflammation, e.g., in the presence of cytokines [28], a new link between inflammation and melanoma has been proposed [58]. In addition, Kong et al. demonstrated that attenuation or deficiency of GC-A expression protect mice from lung, skin (melanoma), and ovarian cancer [59]. It was further reported that ANP prevented metastasis of B16 murine melanoma cells by suppressing their adhesion to the inflamed endothelium [60]. The results suggest that ANP can be considered as a new marker for cancer therapy [61].

An important signaling pathway in melanoma is the RAS/RAF/extracellular signal-activated protein kinase kinase (MEK)/extracellular signal-regulated protein kinase (ERK) cascade [55]. RAS is a small G protein and RAF (ARAF, BRAF, CRAF) is a serine/threonine specific protein kinase. In addition, BRAF is mutated in 50%–70% of spontaneous cutaneous melanoma. The most common BRAF mutation in melanoma (90% of cases with BRAF mutations) involves a glutamic acid substitution for valine 600 (V600E) [62]. Interestingly, Arozarena et al. [63] showed that BRAF^V600E^, acting through MEK and the transcriptional factor BRN2, induce melanoma cell invasion in vitro and in vivo by downregulating the cGMP-specific PDE5A gene, which encodes PDE5. The increase in cGMP due to the repression of the PDE5A gene leads to an increase in cytosolic Ca^2+^, stimulating contractility and inducing invasion. These results suggest that the use of PDE5 inhibitors such as sildenafil (Viagra) in the treatment of diverse disorders (e.g., erectile dysfunction and pulmonary hypertension) may promote malignant melanoma. Importantly, in clinical studies it has been reported that treatment with PDE5 inhibitors caused a modest increase in the risk of melanoma development [64,65]. 

Concerning the role of pGC in melanoma, it has been shown that a large increase of CNP as a ligand of GC-B, which is released by the tumor vasculature particularly under inflammatory conditions, enhances the activity of PKGI in melanoma cells by increasing the intracellular cGMP concentration [66]. Activation of this cGMP pathway promoted mitogen-activated protein kinase (MAPK) signaling, melanoma cell growth in vitro and in vivo, and migration in vitro. These effects were potentiated in the presence of sildenafil. Based on this finding, a model in which CNP acts as fuel and PDE5 as a brake on the growth-promoting cGMP pathway has been proposed [58]. Sildenafil and probably other PDE5 inhibitors release the PDE5 brake, leading to the activation of the cGMP pathway and promotion of the switch from non-metastatic to invasive/metastatic melanoma cells. Indeed, the functional outcome of cGMP signaling depends on its compartmentalization as well as on the expression of the different enzyme isoforms. In addition, two new cGMP analogues were developed that are able to reduce proliferation and migration of melanoma cells in vitro [67]. However, whether such cGMP compounds can be used in the therapy of melanomas, alone or in combination with standard chemotherapy, remains to be investigated. The role of cGMP signaling in melanoma cells is summarized in Figure 2.

## 4. Experimental Approaches to Alter the Influence of Gravity on the Ground 

Long-term experimentation under space conditions is limited due to scare flight opportunities and costs. To some extent the conditions of weightlessness (microgravity) can be simulated on ground. Different principles are in use in order to randomize the effect of gravity [68]. The portfolio of platforms, ranging from ground-based approaches to experiments under space conditions, enables us to alter the influence of gravity and expand our knowledge how gravity has shaped life and influences the physiology of organisms. 

We shortly introduce the ground-based facilities that have been used in the cited literature in this review. The 2D clinostat principle is based on the neutralization of sedimentation, which is achieved by rotation of a test system around a horizontal axis, perpendicular to the direction of the gravity vector. In some cases, a direct comparison with results obtained in real microgravity was already possible and validated 2D clinorotation as an appropriate method to simulate microgravity under limited time frames. A 3D clinostat is characterized by a second rotation axis perpendicular to the other one. Operation is done in a constant or randomly changing mode with respect to speed and rotation direction; the latter one is called “random positioning machine (RPM)”. The rotating wall vessel (RWV) is a further platform aiming to neutralize sedimentation. The 3D-cell cultures are grown in a vessel of several diameters rotated at a speed that avoids their sedimentation.

However, several parameters have to be critically considered to achieve an optimal simulation. Speed of rotation and diameter of exposure determine the amount of residual acceleration. Furthermore, thresholds for gravity sensing as well as reaction time are of relevance, which are, however, in most cases not known. Operational modes, especially changes of rotation directions, might induce shearing forces. Comparative studies between the different simulation approaches are necessary to understand what is really achieved for the exposed systems [68,69]. For example, exposure of a fast biosensor of mechanical stress—the dinoflagellate *Pyrocystis noctiluca*—visualized the shear forces induced by random positioning in contrast to clinorotation [70,71]. It is therefore recommended to perform comparative studies due to the different working principles of the RPM and the clinostat. Furthermore, the quality of simulation should be validated under real microgravity (e.g., in space) [72].

Centrifugation is much easier to apply, as the amount of acceleration is constant and also very effective in identifying the role of gravity on cellular physiology. Centrifuges have been constructed to allow, for example, the investigation of cells under hypergravity conditions [73]. Moreover, centrifugation is an ideal supplement to research under simulated and real microgravity [73], although increased gravitational stimulation provides a completely different condition than complete loss of the stimulus, and furthermore the existence of thresholds reduces the extrapolation of predicted results in microgravity. Dedicated acceleration profiles between microgravity and 1 g can be applied by using centrifuges in space in order to determine thresholds for gravity-related responses. In addition, every space experiment is accompanied by launch and landing conditions and thus increased accelerations, whose impacts on the biological systems have to be known and in consequence separated from the microgravity effects. This makes centrifugation an important method in gravitational biology.

## 5. Differential Effects of Altered Gravity on Guanylyl Cyclase-cGMP Signaling Pathway in Melanocytes and Non-Metastatic Melanoma Cells

The space flight environment (microgravity, cosmic radiation) has many stress factors that may induce different effects on human physiology and health, including reduction in the immune competence in astronauts, leading to an increase in the risk of infection, altered antibiotic susceptibility [74,75,76], as well as a possible development of premalignant/malignant disorders, especially during long-term space flights [77]. In cells grown in space, various changes have been observed, including cytoskeleton rearrangements, altered gene expression, and changes in cell signaling [78,79]. As the GC-cGMP pathway is involved in the melanocyte response to environmental stress and has also been linked to many cellular processes, including melanoma growth and migration, we compared the regulation of the GC-cGMP signaling pathway in a panel of human melanocytes and melanoma cell lines with different metastatic potential and pigmentation in hypergravity as well as under simulated microgravity conditions [80,81,82]. We used a specific centrifuge for the hypergravity experiments (up to 10 g) [80] and a fast-rotating 2D clinostat (60 rpm) as well as a fast-rotating clinostat microscope (60 rpm) to simulate microgravity conditions [81,83]. These devices were manufactured at the German Aerospace Center (DLR), Cologne, Germany.

Cultured human melanocytes and non-metastatic 1F6 melanoma cells, which express functional sGC but low activities of GC-A/GC-B, responded to a long-term exposure to hypergravity (up to 5 g for 24 h) with elevated cGMP efflux in the presence of 0.1 mM 3-isobuthyl-1-methylxanthine, a non-specific PDE inhibitor [80], as well as in the presence of a direct NO donor [84]. Hypergravity also stimulated cGMP efflux in the presence of 1 µM trequinsin, a specific inhibitor of the cGMP-binding PDE5 and of transport by the selective cGMP exporters MRP4/MRP5. Transport was further inhibited by probenecid, an inhibitor of endogenous non-selective transporters as well as of MRP4/MRP5 and by cyclohexamide as inhibitor of de novo protein synthesis. In contrast, hypergravity did not affect cGMP efflux in highly metastatic BLM melanoma cells, which express predominantly functional GC-B/GC-A. This effect was further verified by using a metastatic clone (1F6-m) of the non-metastatic and pigmented 1F6 melanoma cells expressing functional sGC. As expected, hypergravity did not induce cGMP-efflux in the 1F6-m melanoma cells. Thus, hypergravity may stimulate cGMP efflux in human melanocytes and non-metastatic melanoma cells expressing functional sGC via an enhanced expression of endogenous transporter and/or of MRP4/MRP5. In addition, the melanin content as well as the synthesis of cAMP, a well-known modulator of melanogenesis [85], was up-regulated in pigmented melanocytes and non-metastatic 1F6 melanoma cells, indicating a role of cAMP in the hypergravity-induced pigmentation [80].

**Figure 3:**
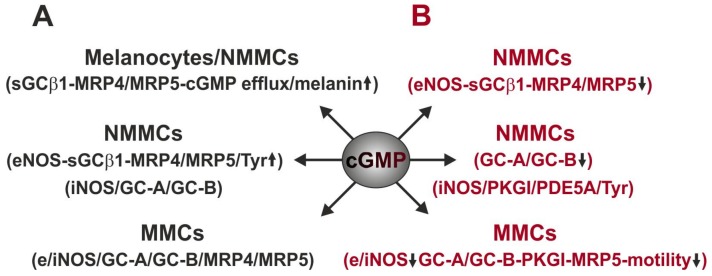
Effects of altered gravity on cGMP signaling in melanocytes and non-metastatic and metastatic melanoma cells. (**A**) cGMP signaling under hypergravity condition (black color). Hypergravity induces an increase in sGCβ_1_-MRP4/MRP5-dependent cGMP efflux as well as in melanin content in human melanocytes/non-metastatic melanoma cells (NMMCs). The expression of iNOS/GC-A/GC-B expression in NMMCs and expression of e/iNOS/GC-A/GC-B/MRP4/MRP5 in metastatic melanoma cells (MMCs) were not altered. (**B**) cGMP signaling under simulated microgravity conditions (red color). Simulated microgravity down-regulates the eNOS-sGCβ_1_-MRP4/MRP5 pathway in NMMCs as well as the expression of e/iNOS, GC-A/GC-B/PKG/MRP5, and motility of highly metastatic melanoma cells (MMCs). The expression of iNOS/PKGI/PDE5A/Tyr in NMMCs was not altered under simulated microgravity condition.

We further showed that hypergravity (up to 5 g for 24 h) was able to induce an increase in the mRNA expression of eNOS, sGC-β_1_ subunit, and MRP4/MRP5 in non-metastatic 1F6 melanoma cells, but there were no apparent changes in the mRNA expression of GC-A/GC-B and iNOS compared to 1 g controls [82]. Thus, the eNOS-sGC-cGMP-MRP4/MRP5 pathway appears to be involved in the hypergravity-induced cGMP efflux in non-metastatic melanoma cells, expressing functional sGC [80]. Moreover, hypergravity was unable to induce changes in the mRNA expression of eNOS and MRP4/MRP5 in non-metastatic melanoma cells transfected with siRNA against the sGC- β_1_ subunit, indicating that a cytosolic-localized pool of cGMP is targeted by hypergravity. Interestingly, the hypergravity-induced increase in the RNA expression of tyrosinase (Tyr, a key enzyme of melanogenesis) in pigmented non-metastatic 1F6 melanoma cells was partly inversed after transfection of the cells with siRNA against the sGC-β_1_ subunit, suggesting a partial role of sGC-cGMP signaling in the hypergravity-induced pigmentation. Thus, hypergravity can be considered as another stimulus that is able to induce an increase in the melanin content in melanoma cells expressing NO-sensitive sGC, which may exacerbate melanoma. The effects of hypergravity on the expression of the investigated cGMP generators and effectors are summarized in Figure 3A.

In contrast to hypergravity, the simulated microgravity (≤ 0.012 g for 24 h) in a fast-rotating 2D clinostat (Figure 3B) down-regulated the eNOs-sGC-MRP4/MRP5 signaling in pigmented non-metastatic 1F6 melanoma cells [82]. In addition, the mRNA expression of PDE5A, PKGI, and Tyr was not changed. Importantly, the mRNA expression of GC-A/GC-B but not iNOS expression was reduced in these cells. Silencing of the sGC-β_1_ subunit reversed the simulated microgravity-induced down-regulation in the mRNA expression of MRP4/MRP5, indicating that down-regulation of MRP4/MRP5 depends on the presence of NO-sensitive sGC. However, as suppression of sGC expression has been postulated as a biomarker for tumor aggressiveness due to the absence of functional sGC expression in many tumors [86], it can be suggested that this type of melanoma cell may undergo malignant transformation during long-term exposure to the microgravity environment.

## 6. Effects of Altered Gravity in Metastatic Melanoma Cells

For the non-sGC expressing human highly metastatic melanoma cells, we reported that hypergravity does not alter the mRNA expression of e/iNOS, GC-A/GC-B, and MRP4/MRP5 [80]. In contrast to the effects of hypergravity, we detected a down-regulation of the mRNA expression of iNOS in highly metastatic BLM melanoma cells under simulated microgravity conditions compared to 1 g. As iNOS expression is absent in benign nevi and present in invasive melanoma [87], and the levels of expression correlate strongly to poor clinical outcome [88,89], the down-regulation of the iNOS expression in the investigated highly metastatic melanoma cells may be involved in the reduction of melanoma aggressiveness in microgravity. Such NO effects would be sGC-cGMP-independent as these cells do not express functional sGC. 

Concerning the molecular player of the pGC-cGMP signaling in highly metastatic melanoma cells, we reported a strong reduction in the expression of both GC-A and GC-B under simulated microgravity conditions in comparison to 1 g controls [82]. In addition, simulated microgravity induced a more potent reduction of the expression of the CNP-sensitive GC-B than of the ANP-sensitive GC-A in highly metastatic melanoma cells. The MRP5 expression was also significantly down-regulated at simulated microgravity (2D clinostat) conditions compared to 1 g, whereas MPR4 expression was not affected. We further observed a significant reduction of the expression of PKGI in the highly metastatic BLM melanoma cells, whereas the PDE5A expression was not changed when compared to 1 g control cells. Finally, we detected that simulated microgravity may reduce the motility of highly metastatic melanoma cells in comparison to 1 g controls [82]. 

For GC-B, it was reported that activation of the CNP-GC-B-cGMP-PKGI pathway in melanoma cells promotes melanoma cell growth and migration in a MAPK-dependent manner [66]. In addition, the amplitude and duration of cGMP signals depend on its rate of synthesis by the guanylyl cyclases, degradation by PDEs, and export into the extracellular space by MRP5. Thus, it can be suggested that the down-regulation of the GC-B-cGMP-PKGI-MRP5 signaling may be involved in the simulated microgravity-induced reduction of motility in highly metastatic melanoma cells. The effects of simulated microgravity on the expression of the investigated player of the cGMP signaling pathway are summarized in Figure 3B. Indeed, not all melanoma cells express the growth promoting cGMP pathway. On the other hand, the variable effects of cGMP on tumor growth and metastasis (pro- and anticancer effects) may be due to the fact that diverse tumors express different cGMP generators and effectors.

In addition, a reduction in the migration has also been reported for a human adenocarcinoma cell line [90] and human glioblastoma U87 cells during clinorotation (30 rpm) [91] as well as an impaired ability of fibroblasts to migrate in “a modeled microgravity conditions” using a RWV [92]. Moreover, it was reported that random positioning on a three-dimensional clinostat (with 30°/s angular velocity) reduced BL6-10 melanoma cell proliferation, adhesion, and invasiveness in vitro and decreased tumor lung metastasis in vivo via FAK/RhoA-regulated mTORC1 and AMP-activated protein kinase pathways [93]. For the highly metastatic BL6-10 melanoma cells, it was further shown that RPM-exposure reduced the focal adhesions and altered the cytoskeleton and the nuclear positioning, leading to an enhanced cell apoptosis via suppression of the FAK/RoA-regulated mTORC1/NF-kB and ERK1/2 pathways [94].

## 7. Conclusion and Perspectives

In summary, our review demonstrates the differential regulation of GC-cGMP signaling in human melanoma cells with different metastatic potential under simulated microgravity (2D fast clinorotation) in comparison to hypergravity conditions, indicating specific effects of altered gravity on melanocyte (patho)physiology. Importantly, the finding that simulated microgravity down-regulated the expression of the NO-sensitive sGC in non-metastatic melanoma cells, whose expression and activity inversely correlate to tumor aggressiveness, suggests that simulated microgravity may promote the transformation from a non-metastatic to a metastatic phenotype. On the other hand, the down-regulation of the cancer-related gene expression of the CNP-sensitive GC-B/ANP-sensitive GC-A as well as of iNOS and motility of highly metastatic melanoma cells may be related to a reduction of tumor aggressiveness in simulated microgravity. It is important to note that cGMP may also affect different processes in the tumor environment, including angiogenesis, inflammation, and the immune response. In addition, several members of the guanylyl cyclase-cGMP signaling are known as drug targets because their activators are associated with diseases of the cardiovascular, skeletal or intestinal systems. Thus, future experiments in real microgravity may benefit from considering the cGMP signaling pathway as a possible factor in the melanocyte transformation or for other human diseases, including cancer. Furthermore, this pathway bears potential for developing protective measures and countermeasures but also in the medication strategy for humans under extreme conditions, especially for astronauts during long-term space flight missions. 

## Abbreviations

2D	Two-dimensional
AMP	5′Adenosine monophosphate
ANP	Atrial natriuretic peptide
BNP	B-type natriuretic peptide
BRAF^V600E^	oncogenic BRAF^V600E^ variant
cAMP	Cyclic adenosine-3′,5′-monophosphate
CGCs	cGMP-gated ion channels
cGMP	Cyclic guanosine-3′,5′-monophophate
CNP	C-type natriuretic peptide
eNOS	Endothelial NOS
ERK1/2	Extracellular signal-regulated protein kinase 1,2
FAK	Focal adhesion kinase
GC	Guanylyl cyclase
5′ GMP	Guanosine 5′-monophosphate
GTP	Guanosine 5′-triphosphate
HF	Heart failure
Hyper-g	Hypergravity
HNOX	N-terminal heme-containing NO/oxygen-binding domain
iNOS	Inducible NOS
MAPK	Mitogen-activated protein kinase
MEK	Extracellular signal-activated protein kinase kinase
MMCs	Metastatic melanoma cells
MRP4/MRP5	Multidrug resistance proteins 4/5
mTORC1	Mammalian target of rapamycin complex-1
NHMs	Normal human melanocytes
NMMCs	Non-metastatic melanoma cells
NF-*k*B	Nuclear factor *k*-light-chain-enhancer of activated B cells
NO	Nitric oxide
NONOates	A family of direct NO donors
NOS	NO synthase
PAH	Pulmonary arterial hypertension
PDE	Phosphodiesterase
PDE5A	cGMP-specific PDE
pGC	Particulate guanylyl cyclase
RhoA	RAS homology gene family, member A
PKG	cGMP-dependent protein kinase
ROS	Reactive oxygen species
RPM	Random positioning machine
RWV	Rotating wall vessel
sGC	Soluble guanylyl cyclase
Tyr	Tyrosinase
URO	Urodilatin
UVB	Ultraviolet B
